# “Posterior interosseus artery flap for hand reconstruction: anatomical basis and clinical application”

**DOI:** 10.1186/s12891-022-05630-0

**Published:** 2022-07-12

**Authors:** Melad N. Kelada, Rasha R. Salem, Youssef A. Eltohfa, Naser A. Ghozlan, Hassan M. Kholosy

**Affiliations:** 1 Department of Human Anatomy and Embryology, Faculty of Medicine, University of Alexandria, N.B. 15 Hussein kabadaya street from El Bitash main, El Agamy, Alexandria, Egypt; 2 Department of Plastic and Reconstructive Surgery, Faculty of Medicine, University of Alexandria, Alexandria, Egypt

**Keywords:** Posterior interosseus artery (PIOA), Posterior interosseus artery flap- hand reconstruction (PIAF), Dorsal hand defects, Upper limb reconstruction

## Abstract

**Summary:**

Flap options for upper limb reconstruction have increased due to better understanding of its vascular anatomy. The posterior interosseus artery flap (PIAF) is used to cover defects of the wrist, hand, proximal thumb, and first web space. This flap has many advantages but requires good knowledge about the anatomy of the posterior interosseus artery (PIOA) and its perforators.

**Methods:**

Twenty upper extremity cadaveric specimens were injected with red latex, Fine dissection of the PIOA and its perforators took place; the perforators were counted, measured, described and photographed.

Twenty patients with dorsal hand defects, had PIAF. Cases have Post-operative care and followed up for 6 months post-operative.

**Results:**

The PIOA was constant in all cadaveric dissections and gave off 4–8 septocutaneous perforators along its course between the extensor carpi ulnaris (ECU) and extensor digitorum (EDM) muscles.

The mean distance of the distal most perforator in the middle third forearm from the ulnar styloid was 10.39 ± 1.54 cm. The anastomosis between the PIOA and the anterior interosseus artery (AIOA) was there in all specimens.

Venous congestion occurred in 10% of the cases and was managed conservatively. Necrosis of the distal third of the flap was inevitable in one case; excellent results were obtained in the other cases 90%.

**Conclusions:**

The posterior interosseus artery flap is an excellent perforator flap for hand reconstruction preserving the ulnar and radial artery; but it has a possible complications such as venous congestion or partial flap necrosis that could be managed conservatively.

**Level of evidence:**

II.

## Introduction

Flap options for upper limb reconstruction have increased due to better understanding of its vascular anatomy. Previously, random flaps and staged pedicled distant flaps were used to reconstruct the upper limb with unsatisfactory results [1].

The posterior interosseus artery (PIOA) originates from the common interosseus artery and less commonly from the ulnar artery in the proximal forearm [2]. It passes to the posterior forearm compartment and emerges underneath the supinator muscle from 15 to 18 cm above the ulnar styloid [3].

In the proximal forearm, the PIOA runs deep along with the posterior interosseus nerve and courses along the septum between the extensor digiti minimi (EDM) and the extensor carpi ulnaris (ECU) [3].

The perforating branches of PIOA run perpendicular to the skin and fan out in all directions [4]. Usually three perforators can be detected on the dorsum of the middle third of the forearm. The perforators form a suprafascial vascular network after piercing the fascia [5].

At the proximal border of the pronator quadratus muscle, around 3–5 cm from the ulnar styloid it divides into two terminal branches: one for the anastomosis with the anterior interosseus artery (AIOA) and second for anastomosis with the dorsal carpal arch. The AIOA/PIOA anastomosis is the basis of the distally based PIAF [3].

The anastomosis between the AIOA and PIOA found in the distal forearm under the extensor indicis muscle and it is important to be preserved and communicating branches to the dorsal carpal arch should be preserved for better viability of the possible flaps [5, 6].

Along the intermuscular septum, the PIOA gives off 4 to 7 septocutaneous perforators. In the middle third of the forearm it gives off 2–4 septocutaneous perforators including one perforator accompanied by 2 venae comitantes that connect the superficial and the deep venous systems [5].

The posterior interosseus artery flap (PIAF) is a small to moderate-sized fasciocutaneous flap and is used to cover defects of the wrist, hand, proximal thumb, and first web space. It can be raised with a segment of ulna as a vascularized bone graft [6]. It can be raised as a skin, adipofascial, or fascial types [7].

The flap design should be kept below the proximal quarter of the line between the lateral epicondyle and the ulnar styloid to avoid necrosis of the flap. Proximal extension of the flap will be based on a random blood supply [8].

The flap pedicle’s length is about 7.1 cm. The most distal perforator of a medium size in the middle third of the forearm represents the distal flap limit is about 7.4 cm above the distal radio-ulnar joint [9].7–8 cm below the lateral epicondyle is the limit of the flap proximally [10].

### Advantages of PIAF

It does not sacrifice a major artery of the hand [1], has an excellent color, texture, and size match for hand and wrist reconstruction [11], preserves the lymphatics on the volar forearm [12] and it has a reliable vascular anatomy, with its long straight vascular pedicle with a good rotation arc [13].

### Disadvantages of PIAF

The PIOA is close to the posterior interosseus nerve and their separation is difficult it requires good microsurgical techniques to avoid injuring the posterior interosseus nerve and The width of PIAF is limited and the donor site requires a split skin graft if the width is more than 5 cm and It has a tedious dissection [13]

## Aim of the work

The anatomical study aimed to put anatomical basis for the posterior interosseus artery as regards its course, relations and branches especially its perforators as a basis for using the PIAF. The clinical study aimed to assess the PIAF as an option for covering hand soft tissue defect.

### Subjects

The anatomical study was carried out on 20 upper extremity cadaveric specimens without any evident trauma or surgery. The clinical study was carried out on 20 patients admitted to the Plastic Surgery Department at Alexandria Main university hospital in the period from February 2019 to June 2020 with dorsal hand defects.

## Methods

### Anatomical study

Twenty upper extremity cadaveric specimens without any evident scars of trauma or surgery were dissected where after exposure of the brachial artery in the arm by fine dissection a catheter was introduced into the brachial artery, Secured and ligated; Red latex was injected to allow fine, accurate identification of the arterial branches [14].

Fine dissection of the PIOA and its perforators took place; the perforators were counted, measured, described and photographed.

Data was fed to the personal computer and analyzed using the social package for statistical sciences (SPSS/ version 20)^.^

### Clinical study

#### All patients were subjected to the following

History taking, thorough examination and accurate diagnosis of the defect, X-rays, routine laboratory investigations.

All studies was performed according to principles of declaration of Helsinki and approved by the local ethics committee of faculty of medicine, Alexandria university (IRB No: 00012098- FWA No: 00018699) and Informed consent was obtained from every patient and from a parent and/or legal guardian for patients less than 16 years old after detailed discussion of the procedure.

**Inclusion criteria were** Patients with dorsal hand soft tissue defects or contractures down to the metacarpophalengeal joints including the first web.

**Exclusion criteria were** Defects can be managed by simpler methods of reconstruction such as skin grafts or need more sophisticated reconstruction such as free flaps, Extremes of age, Smoking, Co-morbidities and injury to the flap pedicle.

#### Surgical procedures

**Fig. 1:**
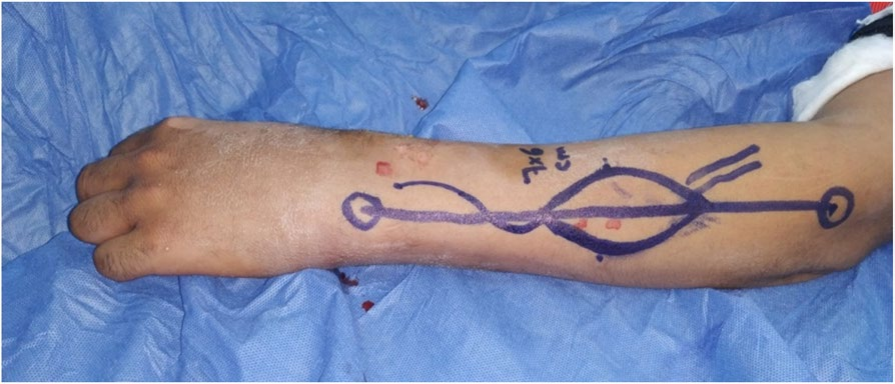
A photograph showing PIAF preoperative markings

Preoperative markings: The required flap dimensions were determined using a cut-to-fit template of the defect and the required pedicle length was measured from the pivot point 4 cm above the ulnar styloid. (Fig. 1).

The design of the flap was centered on the line from the lateral epicondyle to the ulnar styloid. Distal to the midpoint of this line was the middle sized perforator of the PIOA that should be incorporated in the design; its location could be determined by preoperative Doppler sonography. The design of the flap should be 6 cm distal to the lateral epicondyle [15].

#### Operative procedure

The operation was started with superficial incisions on the proximal part of the forearm; flap elevation was started from the ulnar side of the flap. The muscle belly of the ECU muscle was reached, and the deep fascia was incised. The dissection underneath the deep fascia was continued towards the radial side until it reached the perforators coming from the PIOA [16].

The radial side of the flap was then incised through the deep fascia of the EDM muscle, and the flap was raised underneath the deep fascia to the ulnar side until it reached the perforators. A lazy-S incision was made from the proximal part of the flap to a predefined turning point. Flap elevation was started by dividing the vessel in the most proximal part of the flap and then raising the flap, including the intermuscular septum and deep fascia [16].

A wide tunnel was formed to protect the vascular pedicle from pressure then flap was then tunneled to the defect [16].

**Fig. 2:**
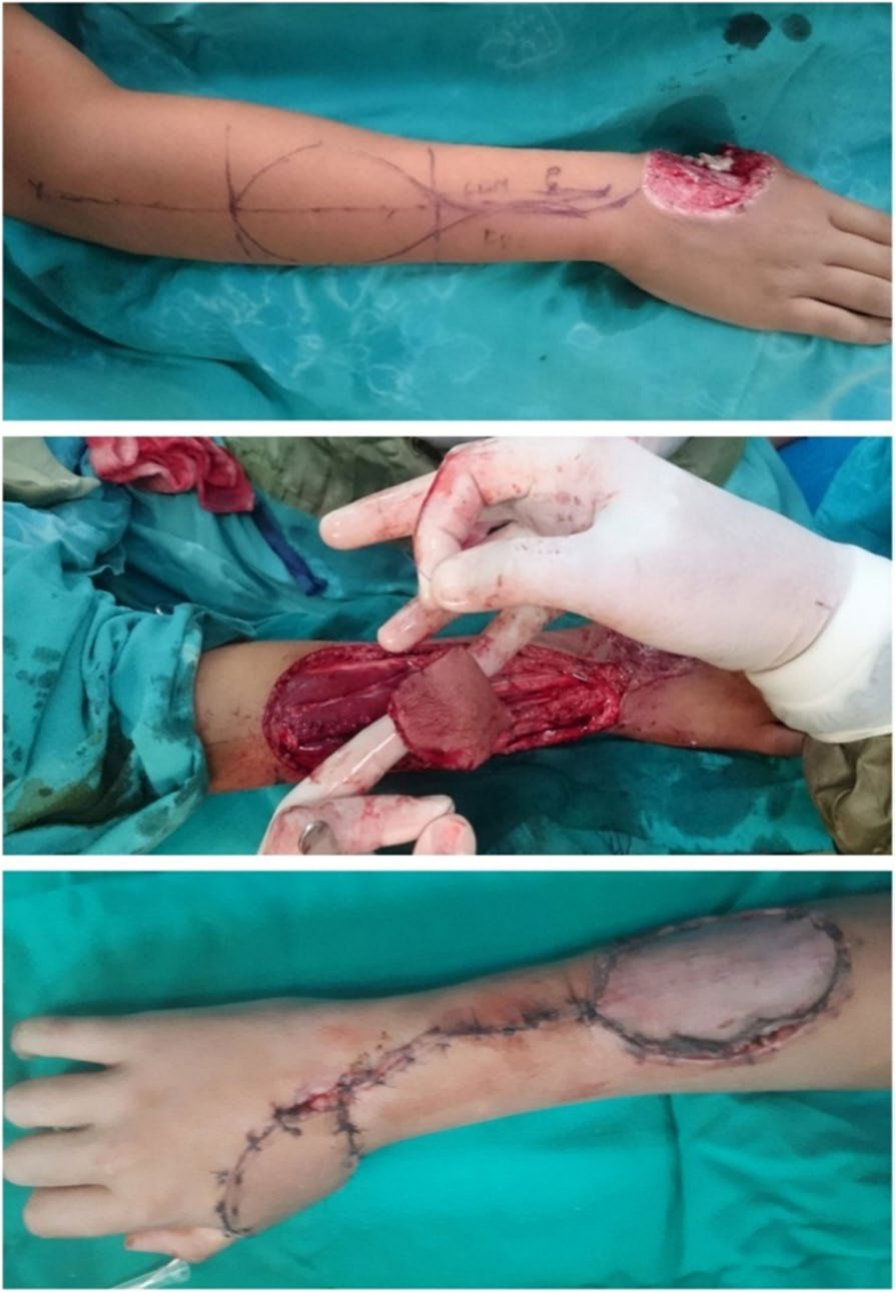
A photograph showing Operative steps of PIAF

The flap was adapted with subdermal and cutaneous sutures. The flap donor area was either directly closed or covered with split thickness skin grafts (Fig. 2) A light wound dressing was applied [16].

Patients were asked to keep their hands elevated for the first three postoperative days. A volar splint blocking wrist flexion was used for 2 weeks post-operative.

### Clinical follow-up and assessment of the results

Patients were submitted to pre-operative and post-operative standard digital photographs of the original defect and flap. Immediate follow up to look for color, capillary refilling, venous congestion, ischemia and hematomas every 2 hours for the first 20 days, then twice a week for 3 weeks for change of dressings and stitches removal, then monthly for 6 months.

## Results

### Anatomical study results

**Fig. 3:**
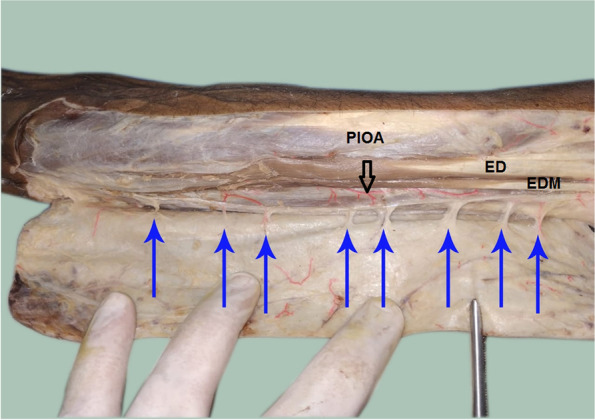
A photograph for the right forearm showing the posterior interosseus artery (PIOA black arrow) giving out 8 septocutaneous perforators along its course (blue arrows). ED = extensor digitorum muscle, EDM = extensor digiti minimi muscle

**Fig. 4:**
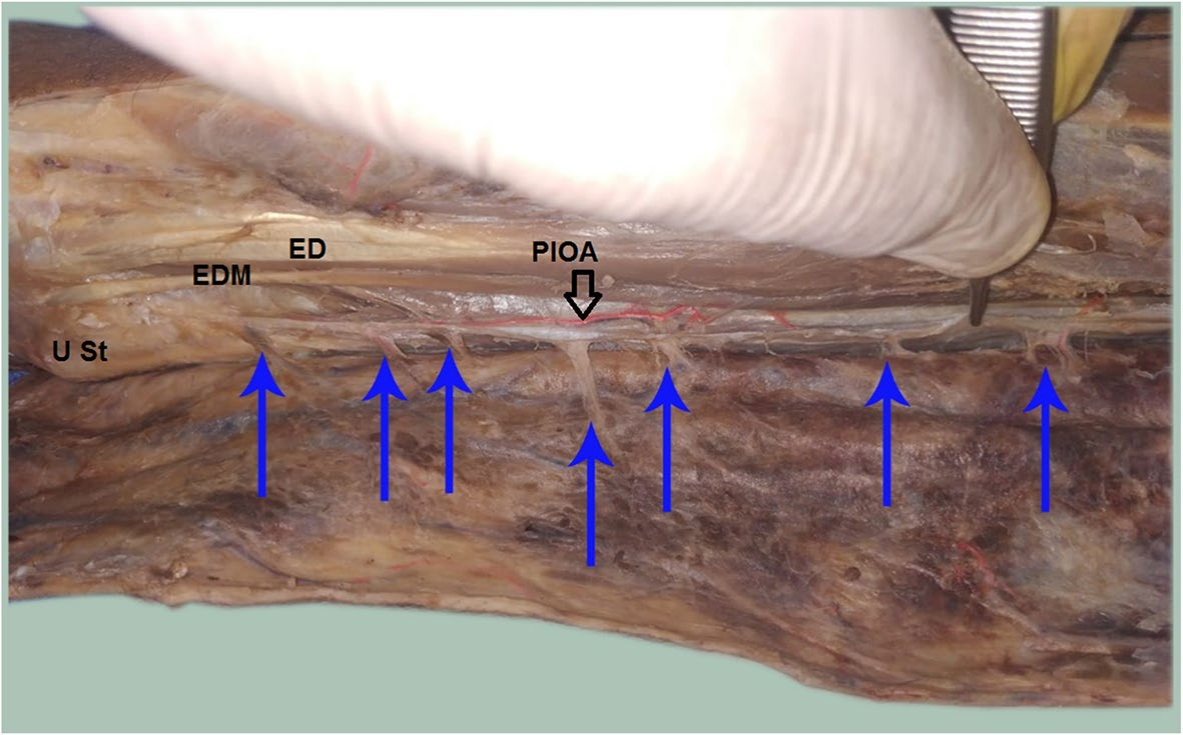
A photograph for the left forearm showing the PIOA (black arrow) giving out 7septocutaneous perforators along its course (blue arrows) ED = extensor digitorum muscle, EDM = extensor digiti minimi muscle, **U St = ulnar styloid process**

**Fig. 5:**
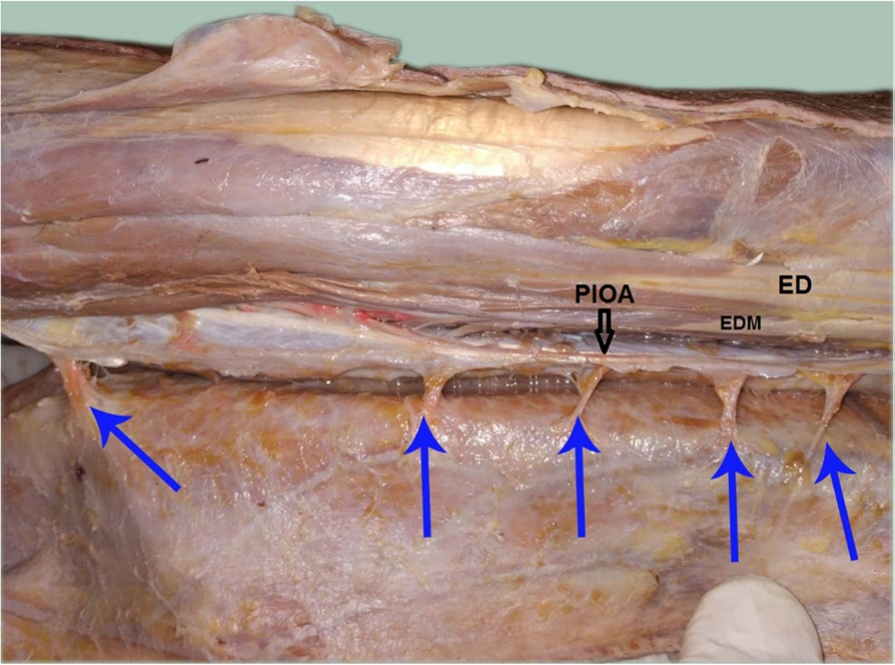
A photograph for the **right** forearm showing the PIOA (black arrow) giving out 5 septocutaneous perforators along its course (blue arrows) ED = extensor digitorum muscle, EDM = extensor digiti minimi muscle

**Fig. 6:**
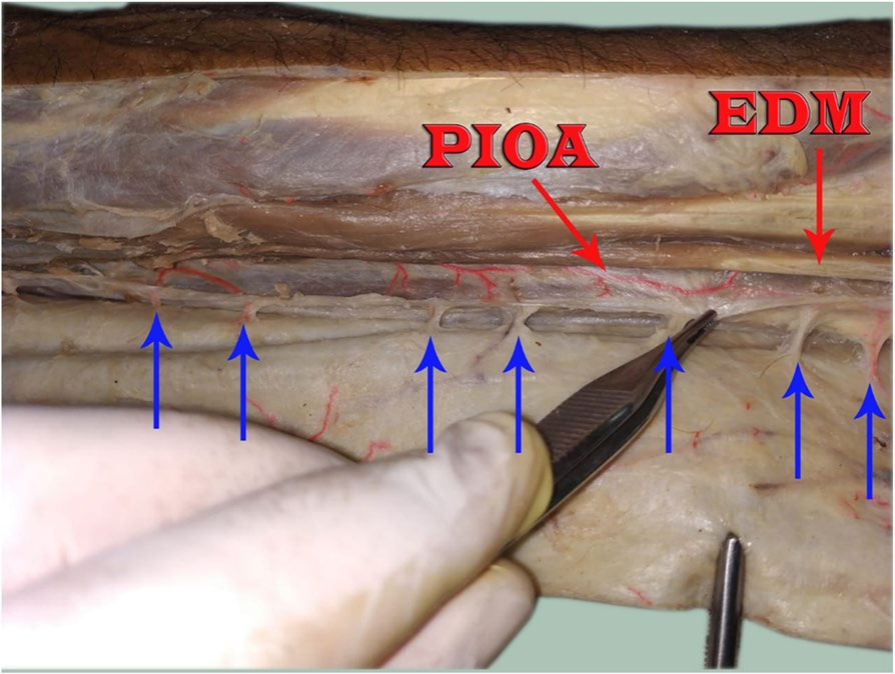
A photograph for the right forearm showing the PIOA giving out 7 septocutaneous perforators along its course (blue arrows), EDM; extensor digiti minimi muscle

The PIOA was constant in all dissections along the whole course. The PIOA gave off 4–8 septocutaneous perforators (mean 5.90 ± 1.33) along its course in the intermuscular septum between the ECU and the EDM muscles. (Table 1, Figs. 3, 4, 5, 6). The number of perforators in the middle third of the forearm was 2–4 (mean 2.50 ± 0.61). (Table 1).

**Table 1:** Distribution of the studied cadaveric specimens according to number of perforators from the PIOA, Number of perforators from the PIOA in the middle third forearm, Distance of the distal most perforator in the middle third forearm from the ulnar styloid (cm), Distance of the communicating artery between PIOA and AIOA from ulnar styloid (cm) and Pedicle length (cm) (*n* = 20)

	Specimens
**Number of perforators from the PIOA**	
Min. – Max.	4.0–8.0
Mean ± SD.	5.90 ± 1.33
Median (IQR)	6.0 (5.0–7.0)
**Number of perforators from the PIOA in the middle third forearm**	
Min. – Max.	2.0–4.0
Mean ± SD.	2.50 ± 0.61
Median (IQR)	2.0 (2.0–3.0)
**Distance of the distal most perforator in the middle third forearm from the ulnar styloid (cm)**	
Min. – Max.	7.30–12.90
Mean ± SD.	10.39 ± 1.54
Median (IQR)	10.60 (9.20–11.30)
**Distance of the communicating artery between PIOA and AIOA from ulnar styloid (cm)**	
Min. – Max.	1.70–3.80
Mean ± SD.	2.87 ± 0.56
Median (IQR)	2.90 (2.45–3.25)
**Pedicle length (cm)**	
Min. – Max.	5.10–9.80
Mean ± SD.	7.52 ± 1.21
Median (IQR)	7.50 (6.65–8.20)

The mean distance of the distal most perforator in the middle third forearm from the ulnar styloid was 10.39 ± 1.54 cm (range 7.30–12.90 cm). (Table 1).

**Fig. 7:**
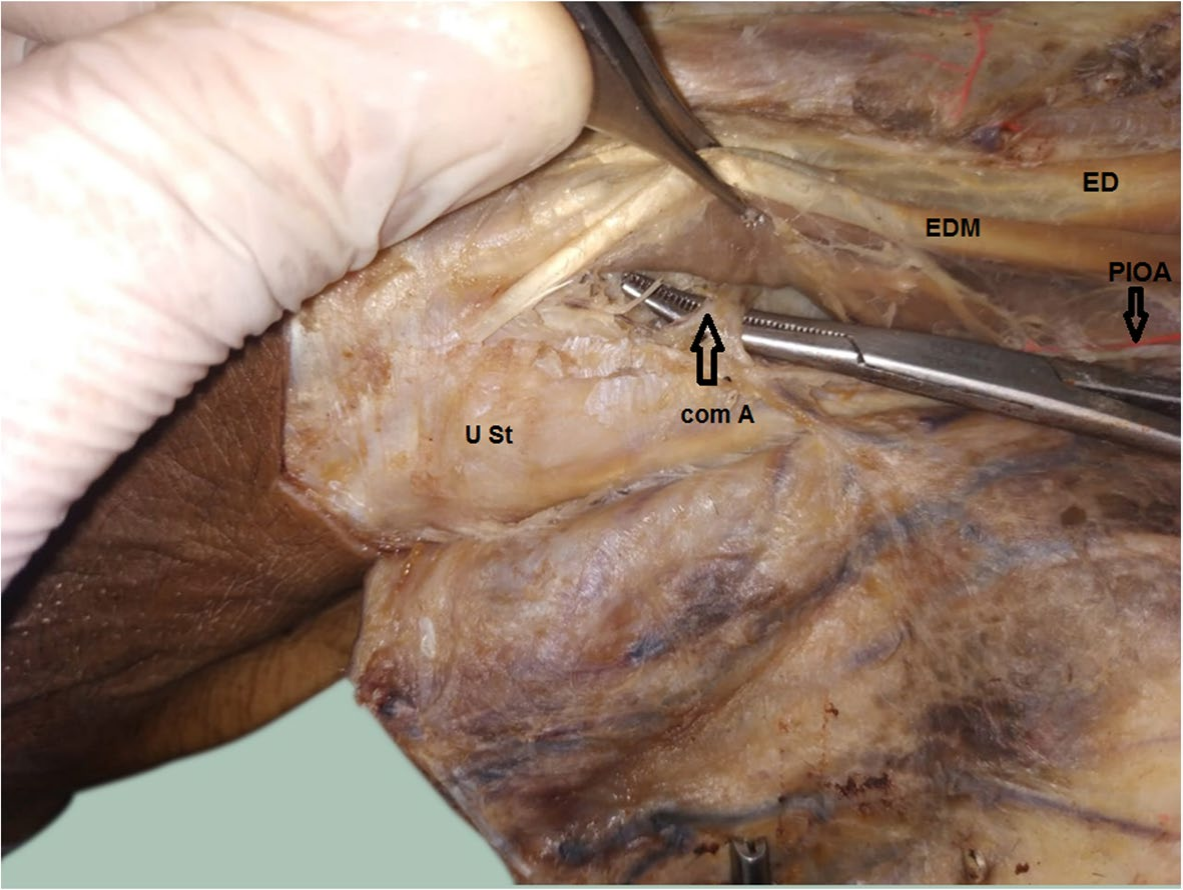
A photograph for the left forearm showing the PIOA (black arrow) and the its communicating artery (com A) (arrow) with the AIOA above the ulnar styloid (U St) ED = extensor digitorum muscle, EDM = extensor digiti minimi muscle, **U St = ulnar styloid process**

The anastomosis between the PIOA and the AIOA was there in all specimens (Fig. 7). The mean distance of the communicating artery between PIOA and AIOA from the ulnar styloid was 2.87 ± 0.56 cm (range 1.70–3.80 cm). (Table 1). The communicating artery located proximal and radial to the ulnar styloid (Fig. 7).

The pedicle length measured from the distal most perforator in the middle third forearm to the anastomosis between the PIOA and the AIOA. The mean pedicle length was 7.52 ± 1.21 cm (range 5.10–9.80 cm). (Table 1).

### Clinical study results

The study was performed on 20 patients two females and 18 males; their mean age was 29.80 ± 12.59 years (range 2–45) (Table 2).

**Table 2:** Distribution of the studied clinical cases according to demographic data, site of the defect, cause of the defect, flap surface area in cm2, donor site closure, operative time in minutes, complications and results

	Conventional PIAF (n = 20)	
	No.	%
**Sex** Male Female	18 2	90.0 10.0
**Age (years)** Min. – Max. Mean ± SD. Median (IQR)	2.0–45.0 29.80 ± 12.59 30.0 (27.0–39.0)	
**Site of the defect** 1st web space contracture Hand amputation stump Dorsum hand	6 4 10	30.0 20.0 50.0
**Cause of the defect** Post traumatic contracture Post burn contracture Post traumatic defect	6 2 12	30.0 10.0 60.0
**Flap surface area in cm2** Min. – Max. Mean ± SD. Median (IQR)	16.50–140.0 65.95 ± 35.03 62.50 (40.0–84.0)	
**Donor site closure** Direct STSG	10 10	50.0 50.0
**Operative time in min.** Min. – Max. Mean ± SD. Median (IQR)	88.0–120.0 100.30 ± 9.91 98.50 (93.0–107.0)	
**Complications** None Venous congestion Partial donor graft loss	18 2 0	90.0 10.0 0.0
**Results** Excellent good Poor	18 0 2	90.0 0.0 10.0

**Fig. 8:**
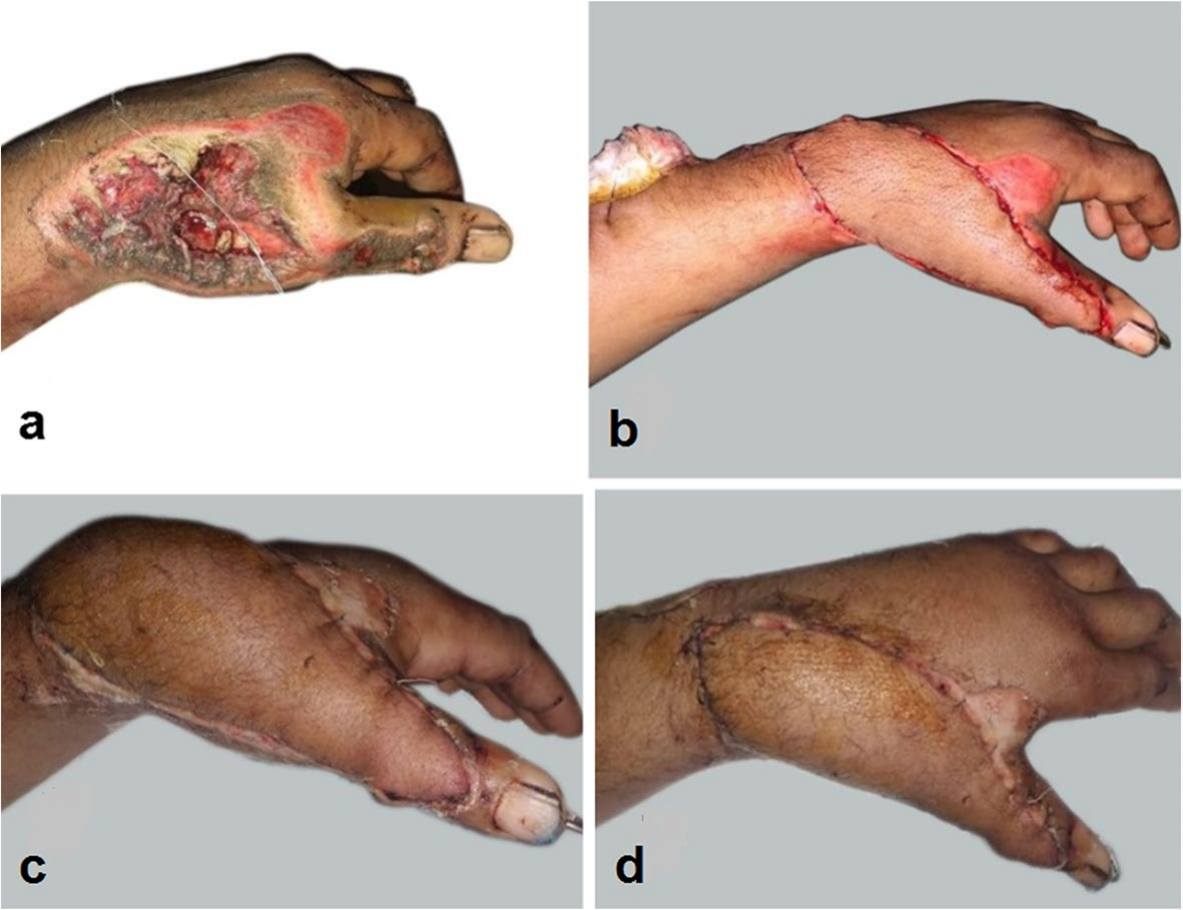
A photograph showing PIAF case to cover a defect on the dorsum of the wrist extending to the dorsum of the thumb (**a**) preoperative (**b**) immediate post-operative (**c**, **d**) 2 weeks postoperative. The donor site was closed with a STSG

**Fig. 9:**
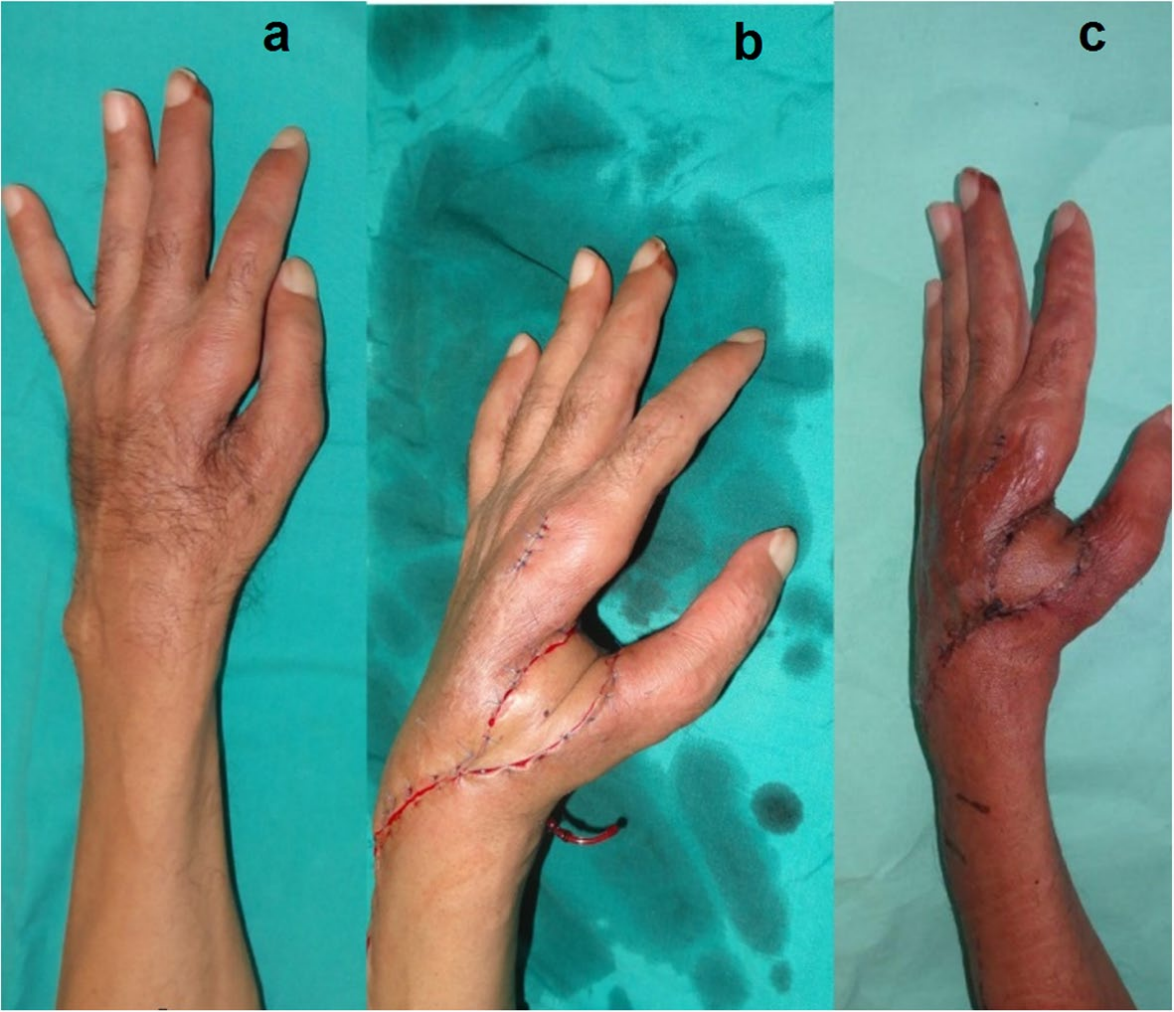
A photograph showing PIAF case to reconstruct a first web space contracture (**a**) preoperative (**b**) immediate post-operative (**c**) one month postoperative. The donor site was closed directly

**Fig. 10:**
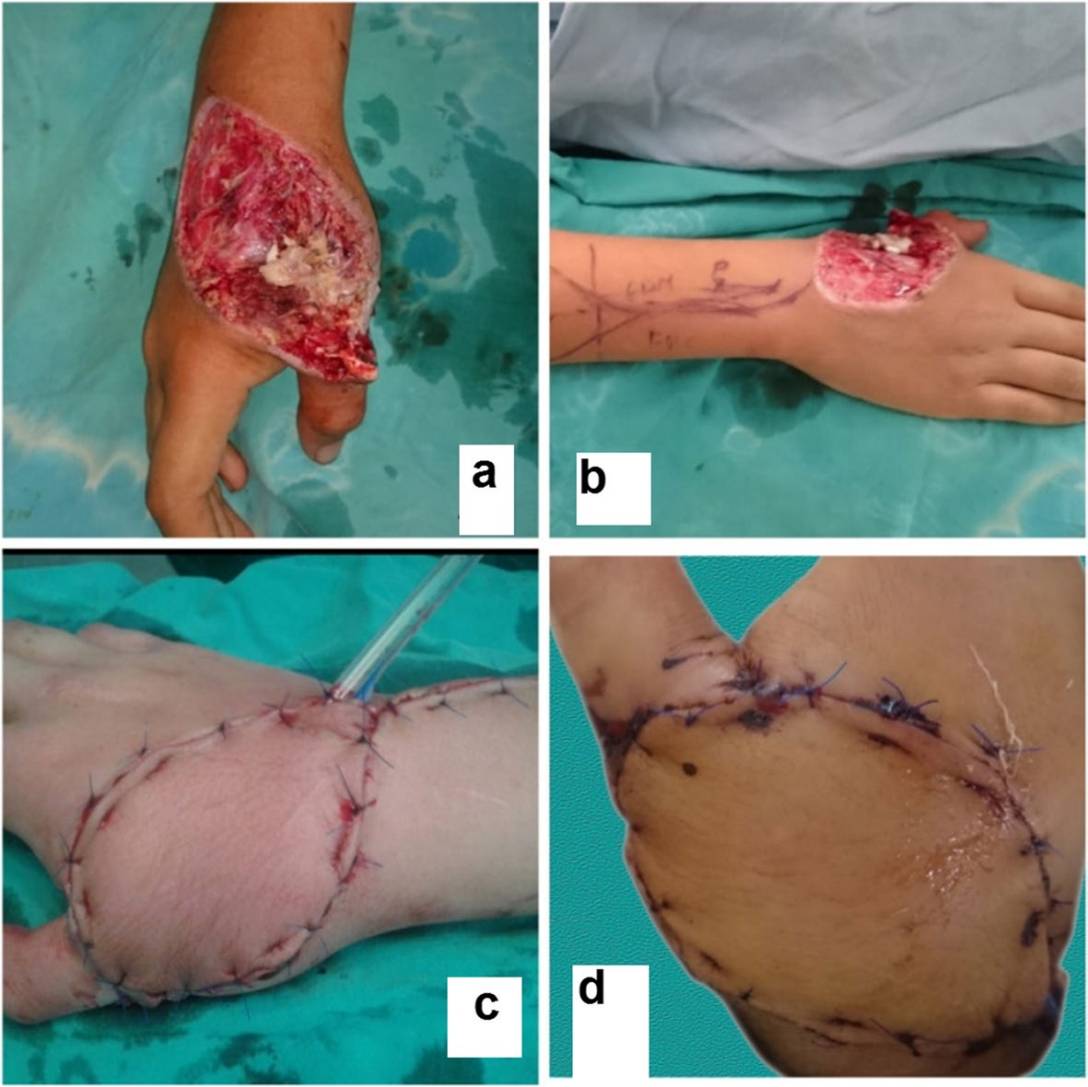
A photograph showing PIAF case to reconstruct a defect involving the dorsum of the hand and wrist (**a**, **b**) preoperative (**c**) immediate post-operative (**d**) 2 weeks postoperative. The donor site was closed with STSG

**Fig. 11:**
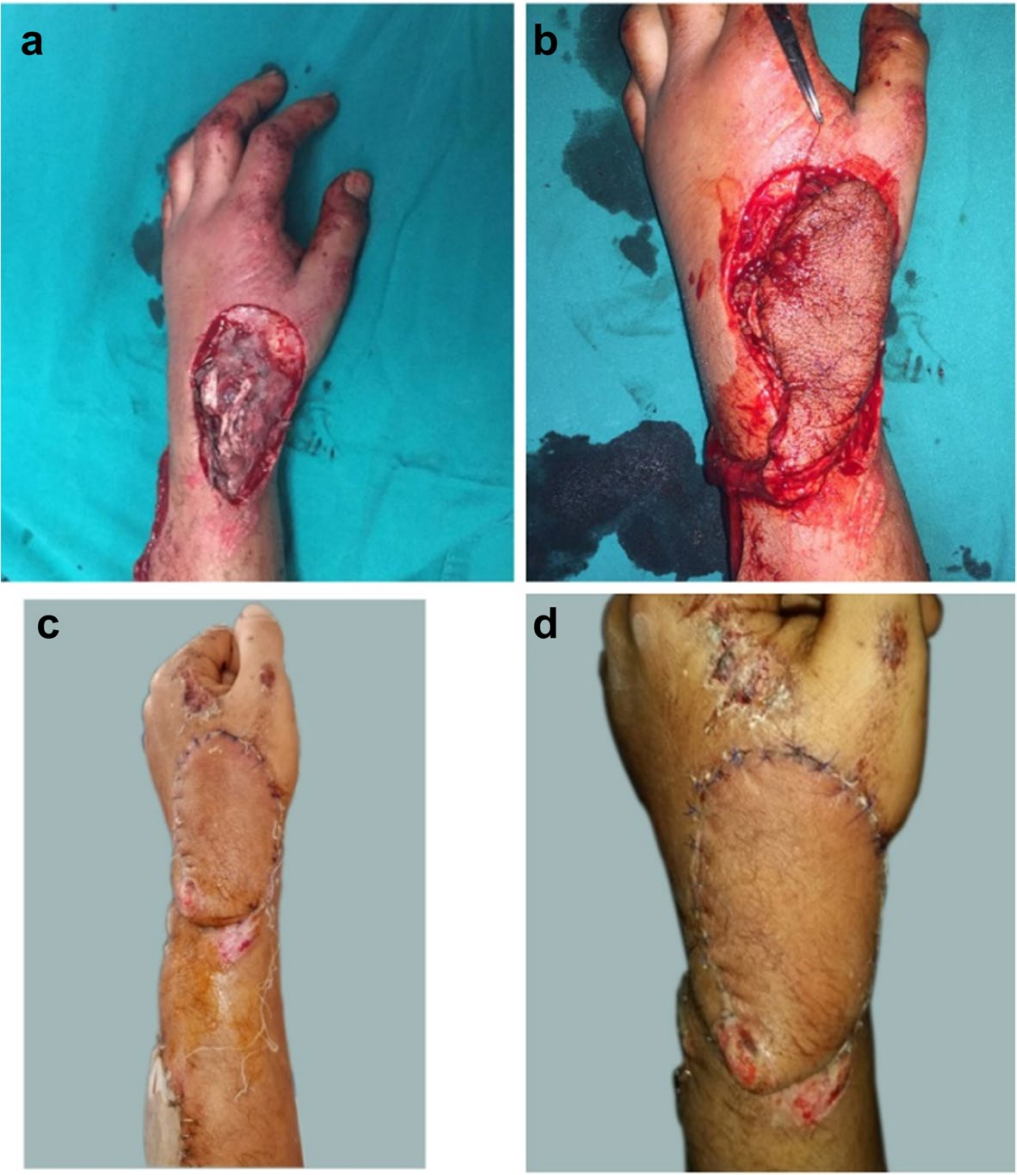
A photograph showing PIAF case to reconstruct a defect involving the dorsum of the hand and wrist (**a**) preoperative (**b**) intraoperative (**c**, **d**) 2 weeks post-operative. The donor site was closed with STSG

**Fig. 12:**
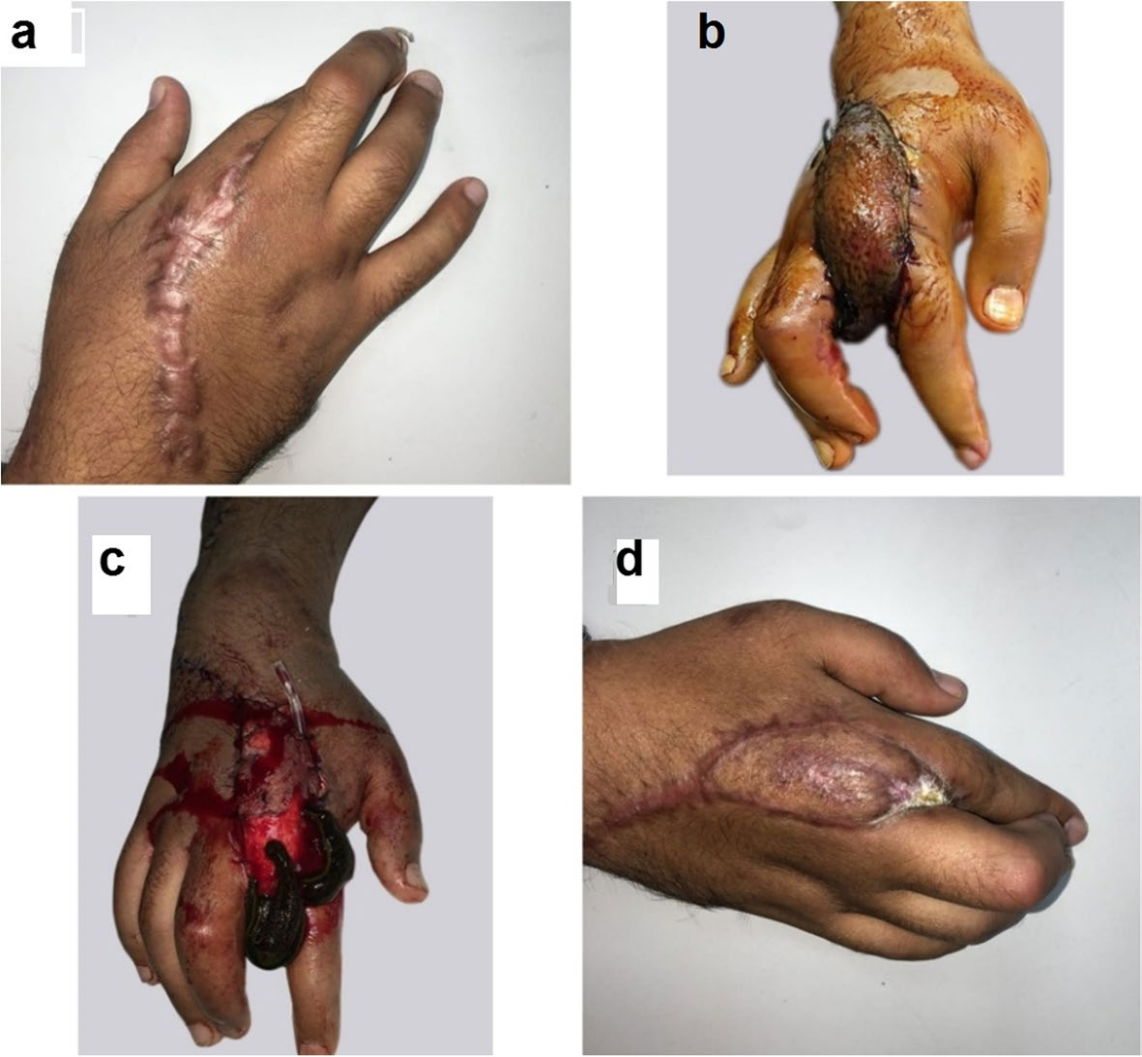
A photograph showing PIAF case to reconstruct a second web space contracture (**a**) preoperative (**b**) post-operative venous congestion (**c**) conservative management by leeches (**d**) distal third flap loss with poor results

**Fig. 13:**
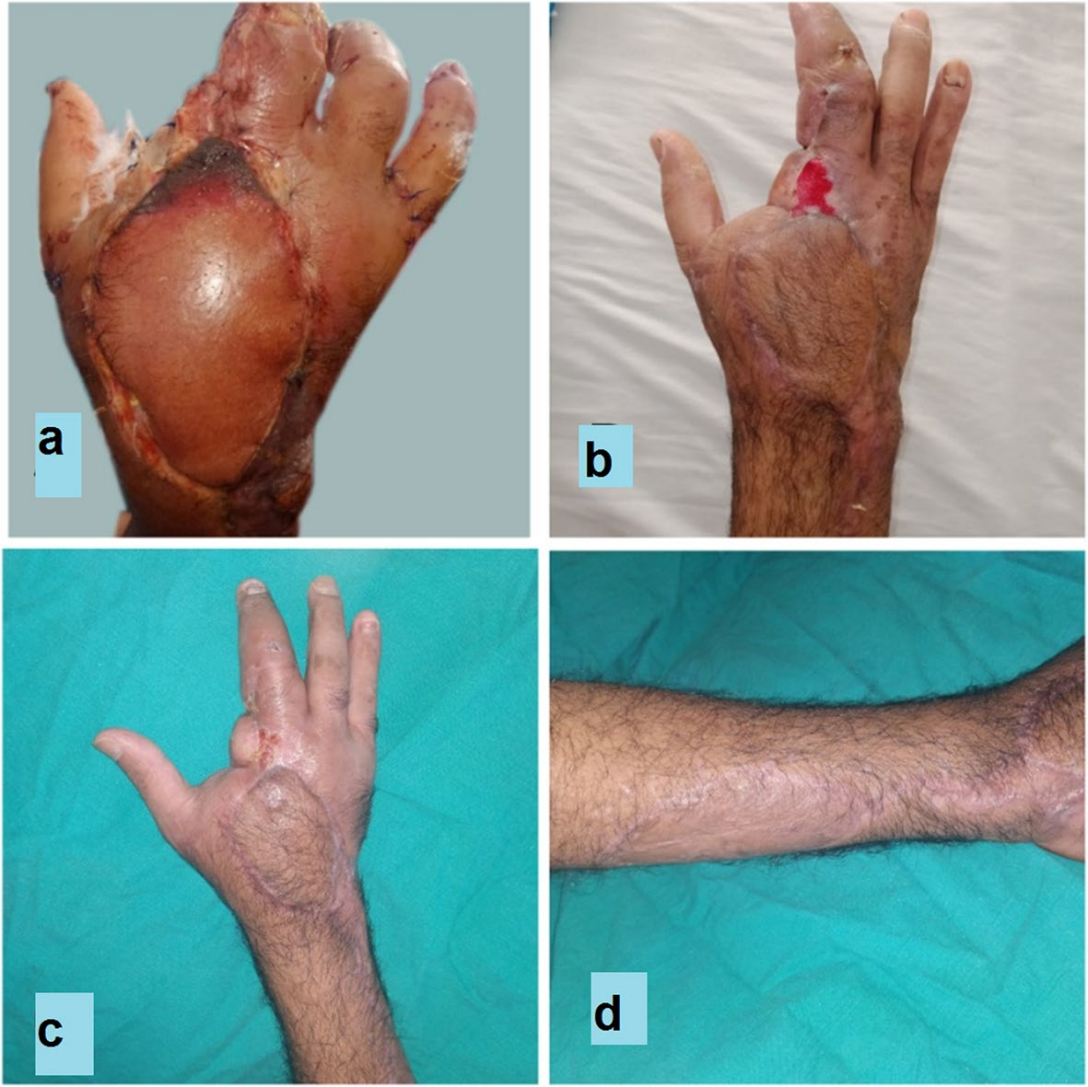
A photograph showing PIAF case to reconstruct a defect involving the dorsum of the hand (**a**) 5 days postoperative showing venous congestion and tip necrosis (**b**) 2 weeks postoperative managed conservatively by repeated change of dressings (**c**, **d**) 2 months postoperative

The sites of the defects were dorsum hand in 10 patients, first web space contracture in six patients and hand amputation stump coverage in four patients. The causes of the defects were post traumatic defects in 12 cases, post traumatic contractures in six cases and post burn contracture in a two cases. (Table 2, Figs. 8, 9, 10, 11, 12, 13).

The mean flap surface area was 65.95 ± 35.03 cm^2^ (range 16.5–140 cm^2^) and the flap width ranged from 3 to 10 cm, while the length ranged from 5.5 to 14 cm (Table 2).

The donor sites closed directly in ten cases where direct closure performed in flaps less than 6 cm in width (Fig. 9). Skin grafts were used in ten cases. (Table 2, Figs. 8, 10 and 11).

The mean operative time was 100.30 ± 9.91 min. (range 88–120 min.). (Table 2).

Excellent results stated in patients who had no or minor complications and did not need any secondary surgical procedures. Good results stated in patients who had venous congestion but could be managed conservatively without secondary surgical procedures. Poor results stated in patients who had venous congestion and needed secondary surgical procedures.

Venous congestion occurred in two cases (10%) and were managed conservatively by leech therapy (Fig. 12) and change of dressings (Fig. 13). Necrosis of the distal third of the flap was inevitable with poor results in one case as the patient needed secondary surgical procedure for second web space reconstruction. Excellent results were obtained in the other 18 cases (90%).

No venous congestion noted when the proximal limit of the flap was distal to the proximal fourth of the forearm and when the PIAF was used for dorsal hand defects down to the metacarpophalengeal joints and first web space.

## Discussion

Major injuries of the hand with composite tissue loss require flap coverage. Early reconstruction of these wounds along with soft tissue repair has become the current standard. The soft tissue repair should be simple, versatile, and safe. The reverse flow PIAF satisfies all of these requirements [17].

### Anatomical study

Twenty upper extremity cadaveric specimens without any evident scars of trauma or surgery were analyzed in this study. The PIOA was constant in all dissections along the whole course.

Lu et al. in 2004 reported a series of 50 cadaveric dissections of PIOA; the PIOA was present in all dissections [18]. In 2007 Costa et al. performed a study based on 100 anatomical dissections of the PIOA. They found that the PIOA present in the intermuscular septum between the EDM and the ECU muscles in all cadaveric dissections which matches our results [19].

Penteado et al. performed 70 cadaveric dissections to describe the PIOA; they reported the disappearance of the artery in the middle third of the forearm in 4 dissections [20]. In a series of 40 fresh cadaveric dissections of PIOA, Angrigiani et al. noted absence of the continuity of the PIOA at the level of the mid-forearm in only one dissection [21].

In this study we found that the PIOA gives off 4–8 septocutaneous perforators with a mean 5.90 ± 1.33 along its course in the intermuscular septum between the ECU and the EDM muscles, 2–4 of them were in the middle third of the forearm with a mean 2.50 ± 0.61.

Lu et al. in 2004 reported a series of 50 cadaveric dissections of PIOA; with average 5–13 septocutaneous perforators. 3–9 perforators with a mean 5.2 found in the middle third forearm, and 2–5 perforators with a mean 3.8 in the distal third forearm [22].

Mei et al. found that the PIOA gave 5 ± 2 septocutaneous perforators [13]. Prasad et al. described 2–4 septocutaneous perforators arising from the PIOA along its course [8]. Sun et al. conclude that the PIOA gave off 6 ± 2 septocutaneous perforators along its course, distributed mainly in middle and distal fifth clusters [18]. Mean number of PIOA perforators was 6 described by Tiengo et al. [23]

The mean distance of the distal most perforator in the middle third forearm from the ulnar styloid was 10.39 ± 1.54 cm and ranges 7.30–12.90 cm in this study. While Prasad et al. in 2014 found it 7.5–10.5 cm [8]. Sun et al. in 2014 found that the main perforator located 6 ± 2 cm proximal to the ulnar styloid [18]. Mei et al. in 2013 reported that the distance between the main perforator and the lateral humeral epicondyle was 11.2 ± 4.8 cm [12].

In this study; the anastomosis between the PIOA and the AIOA was present in all specimens. The mean distance of anastomosis between PIOA and AIOA from the ulnar styloid was 2.87 ± 0.56 cm, ranges 1.70–3.80 cm. The communicating artery located proximal and radial to the ulnar styloid.

Tiengo et al. found that the PIOA and AIOA anastomosis was constant in all 16 specimens [23]. Penteado et al. reported the absence of the PIOA and AIOA anastomosis at the wrist in 1 case out of 70 cases [20].

Lu et al. in 2004 reported a series of 50 cadaveric dissections of PIOA; the anastomosis between the AIOA and the PIOA was present only in 48 specimens, and located 2.5 cm above the ulnar styloid [22].

In the current study; the mean pedicle length was 7.52 ± 1.21 cm, ranges 5.10–9.80 cm.

Sixteen cadaveric dissections were performed in 2016 by Tiengo et al. The mean pedicle length was 10.8 cm. They improved the pedicle length by ligation of the AIOA proximal to the PIOA and AIOA anastomosis; they reported 24% increase in the pedicle length with a mean increase in pedicle length was 2.8 cm; the mean pedicle length after AIOA Section was 13.6 cm [23].

### Clinical study

The main disadvantage of the radial and ulnar forearm flaps include; sacrificing a major artery of the hand which may lead to cold intolerance and varying degrees of weakness, stiffness and sensory loss; so, preservation of these arteries should be considered if there is an alternative flap choice [24].

Many authors reported PIAFs without any major complications; Liu et al. in 2011 performed 26 cases of PIAF for hand reconstruction, all flaps completely survived without any partial or complete flap necrosis [25]. Wang et al. in 2013 performed 13 cases of PIAF to reconstruct dorsal hand defects after sarcoma excision. The mean operation time was 85.77 ± 16.81 min. All flaps survived. Only one case had necrosis of the Z-shaped incision, which managed conservatively [26].

In a trial performed by Tiengo et al. in 2016 to improve the pedicle length by ligation of the AIOA proximal to the PIOA and AIOA anastomosis; they performed 8 PIAF cases. They reported a single case with mild venous congestion with no major complications and increase in the pedicle length to reach down to the PIP joint [23].

Twenty PIAFs were performed in the current study; contained 18 males and two females; their mean age was 29.80 ± 12.59 years (range 2–45). The sites of the defects were dorsum hand in ten patients, 1st web space contracture in six patients and hand amputation stump coverage in four patients. The causes of the defects were post traumatic defects in 12 cases, post traumatic contractures in six cases and post burn contracture in two cases.

The mean flap surface area in was 65.95 ± 35.03 cm^2^ (range 16.5–140 cm^2^), the flap width ranged from 3 to 10 cm, while the length ranged from 5.5 to 14 cm.

The donor sites closed directly in 10 cases; direct closure performed in flaps less than 6 cm in width. Skin grafts were used in 10 cases for donor sites coverage.

The mean operative time was 100.30 ± 9.91 min. (range 88–120 min.).

Venous congestion occurred in two cases (10%) which were managed conservatively by leech therapy and change of dressings. In one case necrosis of the distal third of the flap was inevitable with poor results as the patient needed secondary surgical procedure for second web space reconstruction. Excellent results were obtained in the other 18 cases (90%).

No venous congestion noted when the proximal limit of the flap was distal to the proximal fourth of the forearm and when the PIAF was used for dorsal hand defects down to the metacarpophalengeal joints and first web space.

E. Vogelin et al. 2002 published a retrospective study included 88 PIAF cases done over 15 years; they confronted anatomical variations in 21 cases with failure in flap harvesting in five cases and difficult flap dissection in 16 patients, the rate of complication was elevated due to the anatomical variations. Flap congestion, hematoma, infection occurred with subsequent flap necrosis in 11 cases [27].

### Preoperative investigation

Doppler examination for each patient is necessary to provide information about the course and presence of the posterior interosseus artery and accurate sites of its perforators which will improve pre-operative flap planning and could decrease operative time and post-operative complications [27].

### Surgical planning

The surgical procedure better to start at the wrist to confirm the anastomosis with the anterior interosseus artery and define the transition point of the flap [27].

The vascular pedicle better to be dissected sufficiently wide, if possible including separate veins to decrease post-operative congestion [27].

The cases with the proximal flap extent is far from the perforating vessel, the skin island better to be small with a relatively large fascial flap and this is covered with split skin at the recipient site [27].

### To avoid venous injection

Injection of heparins or enoxaparin sodium 40 units once daily for 5 days could decrease post-operative venous congestion. Intimate post-operative follow up is mandatory as early discovery of the complication results in as fast as possible handling which could results in flap salvage and avoiding bad prognosis [27]. To treat venous congestion, release of tight sutures or leech therapy may be used.

## Conclusions

The posterior interosseus artery flap is an excellent perforator flap for hand reconstruction preserving the ulnar and radial artery. However, it has a possible complications such as venous congestion or partial flap necrosis that could be managed conservatively.

### Limitations of the study

1. Small sample size.
2. Discrepancy between the anatomical studies and clinical situations do exist.
3. Free island flaps may need correction.

## Data Availability

The datasets used and/or analyzed during the current study available from the corresponding author on reasonable request.
